# Understanding consumer beliefs and choices related to food safety: a qualitative study in urban Ethiopia

**DOI:** 10.1017/S1368980024002131

**Published:** 2024-10-23

**Authors:** Stella Nordhagen, Smret Hagos, Genet Gebremedhin, James Lee

**Affiliations:** 1 Global Alliance for Improved Nutrition (GAIN), Geneva 1202, Switzerland; 2 Global Alliance for Improved Nutrition (GAIN), Addis Ababa, Ethiopia; 3 Independent Researcher, Toronto, Canada

**Keywords:** Foodborne disease, Food choice, Traditional markets, Africa

## Abstract

**Objective:**

Provide an in-depth examination of consumers’ food safety beliefs and practices to draw implications for interventions to improve nutrition and food safety in Ethiopia.

**Design:**

Adapted Focused Ethnographic Study approach using in-person semi-structured interviews and free-listing exercises, in two iterative phases.

**Setting:**

A traditional food market in Hawassa, a mid-sized city.

**Participants:**

Forty-six market shoppers, selected randomly in line with quotas for age and gender.

**Results:**

Consumers did not clearly differentiate between quality and safety, seeing them through connected concepts such as ‘freshness’. While most respondents had some understanding of the causes of unsafe food, they did not generally worry about becoming ill themselves and felt food safety risks were easily mitigated through in-home behaviours. Thus, food safety practices were not a main motivator of market or vendor choice. There was no evidence that food safety concerns led consumers to prefer packaged, processed food or to avoid consuming fresh foods.

**Conclusions:**

The study offers novel depth and detail on a topic of strong policy relevance. While building on an encouraging base of understanding of food safety, there remains considerable scope for increasing knowledge, particularly with regard to the need to procure safe food as opposed to expecting household-level practices to mitigate all safety risks. Motivating customers to give food safety factors more consideration when making food purchasing decisions, such as by leveraging emotion-based communication from trusted messengers to elevate the issue’s salience in their minds, may contribute to improvements in food safety in low-income countries such as Ethiopia.

Foodborne disease refers to over 200 illnesses that can be caused by harmful levels of contaminants, such as viruses, bacteria, helminths (worms) and chemicals^(1)^ and is a major public health challenge, particularly in low- and middle-income countries (LMIC). It contributes to an estimated 600 million illnesses and 420 000 premature deaths annually^(1)^, with those living in LMIC comprising about 75 % of deaths (compared with 41 % of the global population)^(1)^. Foodborne disease is closely interlinked with malnutrition: directly, it can increase the risk of undernutrition through reduced appetite, vomiting or diarrhoea, leading to reduced nutrient intake or absorption; increase nutrient needs to recover from illness; affect metabolism; and alter the gut microbiome^(2)^. Indirectly, the desire to avoid foodborne disease can alter consumers’ food choices – which can have nutrition implications since the foods most often implicated in causing illness are highly nutritious animal-source foods and fresh produce^(3)^.

Improving food safety is thus essential for reducing malnutrition and the burden of foodborne illness in LMIC, including Ethiopia, which has high levels of contamination of many food products^(4)^. In the long term, this will require improved regulation and enforcement, but in the short term, Ethiopian government and private-sector capacity to manage food safety in this way is limited^(5)^. Interim approaches are thus needed. This includes influencing consumer demand, which was historically a major driver of safer food in middle- and high-income countries^(6)^. Designing approaches to improve consumer demand for safer food, however, requires understanding the existing motivations and beliefs of consumers; these can then be leveraged and complemented through interventions that enable consumers to demand and choose safer food and take actions to mitigate existing risks^(7)^.

However, the knowledge needed to design such approaches is limited. A 2022 systematic review of food safety research in Ethiopia found extensive prior studies – but almost all of them focused on vendors, with only seven of 119 identified studies focused on consumers^(8)^. Most of these consumer-focused studies focused on knowledge or practices (as opposed to beliefs) and relied on quantitative surveys (‘knowledge, attitude and practice’ surveys) – although it has been shown that qualitative methods have considerable value for examining food safety issues, particularly when it comes to the beliefs and perceptions that shape individuals’ decisions^(9)^. Indeed, ethnographic and in-depth qualitative research on food safety remains relatively rare worldwide^(10)^, with exceptions. Most studies also focused on narrow populations (such as students or mothers of young children), not representing the broader consumer population.

To help fill these gaps, this paper uses data from in-depth qualitative interviews and cognitive mapping techniques to examine the food safety beliefs and practices of consumers in Hawassa, a mid-sized Ethiopian city. We focus on beliefs and practices related to vegetables, as they are highly nutritious but under-consumed in Ethiopia and many other African countries^(11,12)^, are relatively understudied and are prone to food safety risks^(2,4)^. The results are then discussed to draw potential implications for improving food safety in Ethiopia and other LMIC using consumer-focused approaches.

## Methods

This study used a Focused Ethnographic Study (FES) approach, adapted to focus on food safety topics. FES has been applied to various public health nutrition topics^(13)^, including food safety in Nigeria^(14)^. FES employs ethnographic research methods, such as in-depth interviews and cognitive mapping. However, it applies them in a targeted manner to investigate specific inquiries. Its objective is to provide a comprehensive understanding of the behaviours, beliefs and environmental factors, both physical and social, which influence the subject under examination (here, food safety). Research participants shape the research direction through their responses, leading to various follow-up questions and potential areas of focus. The initial phase aims to identify primary themes, while the second phase validates and delves deeper into these. FES was considered appropriate for use here due to our interest in both in-depth understanding of community members’ perspectives under an interpretivist paradigm (the ‘ethnographic’ aspect of FES) and the desire to provide specific and actionable insight on a narrow topic (the ‘focused’ aspect of FES). This specific focus is achieved through more narrow questioning than would be the case in a typical ethnographic study, a more temporally condensed approach and including questions designed to unearth actionable information (as opposed to general knowledge).

Data collection focused on the traditional open-air food market in Hawassa, a mid-sized city in southern Ethiopia. Mid-sized cities are an appropriate focus as they tend to receive less research, despite collectively containing large populations, are different from large capitals (i.e. Addis Ababa) in terms of infrastructure and face particular food safety challenges, and their markets support large shares of the surrounding population^(15,16)^. Within Ethiopia, Hawassa is roughly the same size as several other main secondary cities but faces fewer security concerns, making it a more feasible location for research and intervention. Traditional markets play an essential role in food security and nutrition in LMIC^(17)^ but also face challenges in controlling foodborne pathogens due to inadequate infrastructure^(18)^, hygiene and storage conditions and oversight/regulation issues^(19)^.

The study employed two methods for gathering data: semi-structured interviews and free-listing. During the free-listing process, participants were requested to name all the items belonging to a specific category (e.g. ‘foods that can generally be considered safe’). This information was then compiled to generate a comprehensive, aggregate list representing the population. Items mentioned frequently and early in the process were deemed particularly significant. Semi-structured interviews were conducted using a comprehensive interview guide, drawing on those used in prior studies^(9,14)^, while allowing interviewers the flexibility to diverge from it based on participants’ responses. To ensure the capture of key contextual issues, interviews examined shopping- and food-related concerns broadly before delving into food safety, considering first the general concept and then specific foods and behaviours. In line with FES’s iterative approach, the Phase 2 interview guide was developed based on the results of Phase 1, with the aim of delving deeper into emerging issues from Phase 1, or topics covered in Phase 1 but on which more detail was needed.

Participants were recruited at Hawassa’s main market using random sampling that adhered to predetermined quotas based on age group and gender (half men, half women, half under age 30, half over age 30, chosen as the dividing line between ‘younger’ and ‘older’ adults due to Ethiopia’s overall very young population) to obtain a population that was roughly representative of Hawassa adults. The Phase 1 (June 2022) and Phase 2 (August 2022) sample sizes were sixteen and thirty consumers, respectively, for a total sample size of forty-six. Sample sizes were chosen to achieve saturation of viewpoints and are in line with normal sample sizes for in-depth ethnographic studies^(20,21)^; they appeared adequate, as there was a considerable amount of convergence on similar opinions within the sample, even in Phase 1. Interviews were conducted in Amharic by trained local interviewers with prior experience conducting qualitative research. Interviews lasted approximately 60–90 min. They were audio-recorded, transcribed verbatim and translated into English. Demographic data were analysed using Stata SE15. Anonymised interview transcripts were subjected to thematic analysis following the six-phase framework of Braun and Clarke^(22)^ and using the qualitative data software ATLAS.ti. This began with a list of codes identified *a priori* based on prior research (and in the case of Phase 2, codes used in Phase 1); this code list was added to iteratively throughout the coding process. After coding, codes were collated into themes, and all data on a given theme were viewed jointly to define key findings within that theme. Key findings are illustrated with anonymized quotes in the text; unless otherwise noted, these represent typical views among respondents. Free-list data were analysed using the approach of Weller and Romney^(23)^ via Visual Anthropac 4.9 software. Emerging results were triangulated through two presentations to experts in food safety and/or behaviour change, both Ethiopian and foreign; a comparison with (unpublished) data from the same market collected via a cross-sectional survey and through a rapid field experiment; and a stakeholder workshop involving Hawassa consumers, vendors, educators and government actors.

The study was conducted based on a detailed protocol developed and approved by an institutional review board prior to data collection, and reporting is consistent with the Standards for Reporting Qualitative Research. All study respondents were explained the procedures, risks and benefits and provided informed consent prior to data collection. All data were treated with strict confidentiality and anonymised prior to analysis. Considering researcher positionality, the research team was diverse in terms of gender and age; two researchers were Ethiopian, while two were from high-income countries, thus bringing divergent views to the topic. All of the researchers were purchasers of food, and several of them have purchased from local markets such as those studied. However, all were wealthier and with access to more information and education than most members of the studied population.

## Results

The demographic characteristics of the consumers included in the study are summarised in Table 1. This includes all consumers interviewed in Phases 1 and 2, including those who only responded to the free-listing questions in Phase 2. The following sections first discuss general shopping practices to provide context, then consider motivations for choosing certain markets and vendors (including but not limited to food safety) and then delve into food safety in detail.

**Table 1.** Consumer demographic characteristics (*n* 66)

Respondent characteristics		Household characteristics	
Gender	Male (50 %), female (50 %)	Avg. household size (range)	4·0 (1–10)
Median age (range)	30 (18–55)	Home has electricity	100 %
Main language	Amharic (61 %), Sidama (15 %), Wolaitta (13·6 %), Kebanta (4·6 %), others (6 %)	Household owns refrigerator	41 %
Pct. completing primary school	95 %	Household owns car	1·5 %
Pct. completing tertiary school	47 %	Household owns radio	62 %
Marital status	Married (monogamous) (61 %), single (35 %), divorced or widowed (4 %)	Household owns TV	83 %
Occupation	Professional/managerial (36 %), not employed outside home (21 %), unskilled labour (9 %), others (34 %)	Household owns computer	32 %
		Household owns mobile phone	100 %
		Household has improved toilet	67 %
		Household is engaged in agriculture	6 %
Respondent is household’s principal income earner	65 %	Pct. poor (1·90 PPP)*	4 %
		Pct. poor (3·10 PPP)*	17 %

* Likelihood of living in poverty is calculated via the Poverty Probability Index (https://www.povertyindex.org/about-ppi), using a threshold of 1·90 or 3·20 Purchasing Power Parity (PPP)/person/d.

### Shopping practices: the traditional market is a key food source, despite inconveniences

Shoppers typically go to the market 2–3 times a week, favouring the main market days when availability is better and products are thought to be fresher. Consumers generally buy vegetables each time, and purchases were generally planned in advance but adjusted upon arrival based on market availability and price. The traditional market was the main food purchase location, but some used local shops and mobile neighbourhood vendors, including for vegetables. These local shops or mobile vendors were primarily chosen for convenience: purchasing small items between trips to the main market saved travel time and cost of transportation. There were some inconveniences associated with shopping, particularly the crowded, hectic nature of the market and it being dusty or dirty, especially during rainy periods when it became muddy; a few respondents also mentioned theft, overcrowding and a lack of shade. Women played a larger role in shopping than men, but men were also involved; women were exclusively responsible for cooking.

The main reasons for choosing to shop at the studied market were price, availability, quality (namely, freshness) and convenient location. Respondents appreciated being able to get all items in one place and to have a sufficient number of vendors that they could compare shops among them. It was also widely recognised that prices in neighbourhood shops tended to be more expensive than in the market and might not have as fresh products. Food safety and cleanliness were not mentioned as a motivator of market choice, but some respondents noted that the market was often unclean.

My reason is, of course, the price and the quality. You also get fresh ones from there. At the nearby market, you get stale vegetables since they bring it from the market and sell it. But at the market, if you don’t like something, since it’s big, you have the option to go around and buy a good one. (Female consumer, 1207)

### Choosing a vendor: routine, price, quality and politeness – but rarely cleanliness

Most respondents had ‘regular’ vendors to whom they went repeatedly, at least for certain foods. But nearly all also expressed some flexibility: if their chosen vendor did not have what they were seeking or did not have a competitive price-and-quality combination, they would go to another. Repeat customers noted that going to the same vendor would often get them some special treatment, in terms of product quality, pricing/discounts and other services, such as credit. Several customers, however, noted that they chose to switch vendors regularly to avoid being cheated on prices.

The main reasons cited for choosing a vendor were quality, price and niceness/politeness. Most respondents named at least two of these reasons, and price and quality were seen as somewhat interrelated and, for many, as necessary conditions: in particular, several suggested that there was a minimum sufficient quality that food needed to have before the other aspects came into play.

Neatness or cleanliness was mentioned as a factor in vendor choice by only a few consumers and usually just in passing and not at length; these respondents referred to the produce being neatly arranged (e.g. in a bowl or on a platform as opposed to in the dirt) or physically washed or cleaned as an attracting feature of a particular vendor. As atypical examples, one consumer explicitly connected quality to safety (see quote from 1201, below) and another to health (1207, below), but for the majority, this linkage was not made (at least not explicitly, without prompting). In both market and vendor choice, the most obvious manifestation of potential food safety risk – vendor or food cleanliness – was not a prominent factor for most consumers, and very few made connections between food market or vendor choice and food safety or health.

Quality is the first thing. … Quality for me is ‘life’. It is life, and it is because I don’t spend much on my health. My kids have never complained about a stomachache, let alone me… There are times when she [my wife] shops that the kids complain of pain… So, I have told her to shop only from a certain area. (Male consumer, 1201)

I go to her [my usual vendor] because… her price is good, and her product is fresh. Since it’s good for my health and my family’s health, I always buy from her. Another thing I like about her is her greeting. … it makes me feel like we’re family, and it makes me happy to buy from her. (Female consumer, 1207)

### Distrust of vendors is widespread

Consumers generally reported distrusting vendors and suspected them of many types of unscrupulous behaviour: not weighing accurately; mixing goods of different quality together and selling at the price of the higher-quality product; selling products with grass, chaff or dirt mixed in; using attractive items to hide damaged ones, but selling all at the same price; selling spoiled, damaged or insect-infested foods; charging prices higher than the current market price; and selling old or non-fresh foods without disclosing this. Nearly all respondents named at least one of these behaviours. The possibility of this behaviour for some made it more important to rely on a few trusted vendors – while for others, it made them more motivated to ‘shop around’ to ensure they were not being cheated.

[Vendors] don’t weigh the item properly when you buy… I don’t trust them because of this. They don’t place the unappealing ones up front. They put the skinny ones and the rotten ones on one side and place the good ones in the front so they can attract people… [then] they just gather up [the vegetables you ask for] from the back [where quality is bad]. (Female consumer, 1214)

### Food safety: mixed levels of understanding

Just over half of respondents clearly understood ‘food safety’ as being in line with the common scientific meaning (i.e. food was made unsafe by being contaminated, prepared unhygienically, spoiled or expired); such ‘standard’ understandings are illustrated in the quotes below. Others associated it with other aspects of healthiness (e.g. non-communicable disease prevention, nutrition, dietary diversity), with food security or preferences or with completeness of a recipe or did not know/understand the term at all. Several who identified aspects related to contamination or hygiene combined that with other aspects, such as nutrition. Foodborne disease was universally associated with gastrointestinal symptoms (stomachache, diarrhoea, vomiting); other symptoms were only rarely mentioned, with none referring to non-acute illness (e.g. aflatoxin contamination).

Well, food safety means when you become exposed to typhoid by eating prolonged food… It starts from the food making. If you use a spoiled food when cooking, then it can make whole food you are making unsafe. … the person making the food must wash his hands; the knife and the chopping board must be washed. All the utensils must be washed. If not, the food that is about to be cooked will be easily spoiled. (Female consumer, 1205)

We say food is safe in the way it is handled if it is stored in its appropriate place. Then, the way it is washed or prepared should be attractive and also be prepared with clean materials. If this is done, I consider that this food is safe. It has no harm, it is safe and has no harm to health. (Female consumer, 2252)

**Figure 1.**
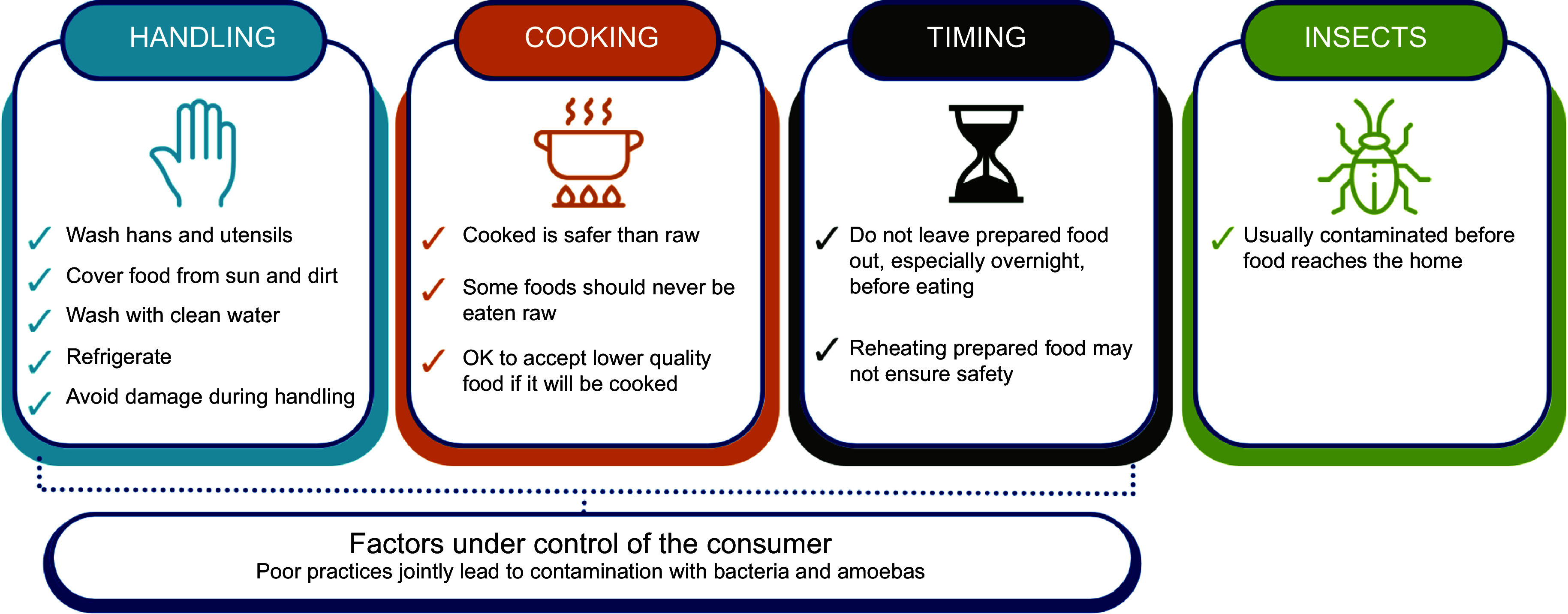
Respondents’ views on factors influencing food safety.

At the same time, most respondents understood many of the mechanisms shaping food safety. In particular, consumers saw three key causes of food becoming unsafe: poor handling or storage; food not being properly cooked or being eaten raw; and spoiled leftover food. These practices were seen as being connected to contamination with bacteria, or to a lesser extent amoebas, which led to unsafe food. All of these factors were under the control of the consumer, whereas insect damage/infestation was also cited but generally occurred before food reached the home/market. There was thus a clear mental model of unsafe food causes and how those related to consumers’ nexus of control, as depicted in Fig. 1. More rarely named causes were production in an unclean environment, contamination with chemicals (e.g. pesticides), physical contaminants such as stones and packaged food that was past its expiration date.

### Food safety is situational, not absolute

Through these remarks, it was clear that food safety was seen as situational (i.e. related to the situation within which a given food was prepared or consumed) not absolute (i.e. a property of a given type of food). Food safety being situational was confirmed by the free-listing results (Tables 2 and 3), which showed a fair amount of overlap between foods respondents named as ‘safe’ and ‘unsafe’. Only lettuce, milk and leftover food stand out as being frequently named as ‘unsafe foods’ but not also named as ‘safe foods’, while only cooked lentils stand out as frequently named as ‘safe’ but not also named as ‘unsafe’. This is in contrast to free-listing results from similar studies elsewhere, which uncovered clear associations between specific foods and safety^(14)^.

[Bacteria and amoeba contamination are] due to [poor] hygiene. If you want to prepare it chopped and raw, it doesn’t get heated. So, both your hands and the things you handle need to be washed well. The problem may not be with the tomatoes, but with the people. For example, the reason one gets sick when they eat raw tomatoes may be due to a problem with the person who serves them. One must check if the hands, the knife, the chopping block, and the lemon to be added are washed thoroughly. If they eat like that, I think it doesn’t cause illness. (Female consumer, 1222)

After we bring it [food] home, if we don’t wash it, I would say it’s unsanitary. So, we need to wash and use what we buy from the market… it comes from different places, and when you go to the market, you see cars, people walking about, dust, and a lot of bacteria that we don’t see with our naked eye. (Female consumer, 1202)

**Table 2.** ‘Unsafe foods’: free-listing results with consumers (Phase 1; *n* 16)

Food	Frequency	Avg. rank	Salience
Kale	50 %	2·75	0·32
Tomato	50 %	2·13	0·369
**Leftover food**	31 %	3·4	0·206
Cabbage	25 %	2·75	0·17
**Lettuce**	25 %	2·75	0·146
**Milk**	19 %	3	0·105
Potato	19 %	3·67	0·096
Shiro (chickpea stew)	12·5 %	4	0·052
Salt	12·5 %	2	0·083
Meat	12·5 %	1	0·125
Raw meat	12·5 %	3·5	0·056
Vegetables	12·5 %	5	0·067
Banana	12·5 %	5	0·033
Food from outside the home	12·5 %	4	0·061
Avocado	12·5 %	5	0·035

Note: Items named by only one respondent are omitted. Those named by more than two people and not also named as ‘safe’ food are in bold text.

**Table 3.** ‘Safe foods’: free-listing results with consumers (Phase 1; *n* 16)

Food	Frequency	Avg. rank	Salience
Potato	25 %	3	0·173
**Miser wot (lentil stew)**	25 %	3·25	0·146
Meat	25 %	1·75	0·198
Egg	18·8 %	5·33	0·046
Shiro (chickpea stew)	18·8 %	2·67	0·135
Tomato	18·8 %	1·67	0·156
Beans	12·5 %	5·5	0·039
Oranges	12·5 %	3·5	0·047
Kale	12·5 %	3	0·076
Injera	12·5 %	2	0·1
Sweet potato	12·5 %	5	0·04
Pasta	12·5 %	2·5	0·09
Cooked meat	12·5 %	3	0·066
Bread	12·5 %	2	0·083
Cooked tomato	12·5 %	3·5	0·065
Cabbage	12·5 %	3·5	0·047

Note: Items named by only one respondent are omitted. Those named by more than two people and not also named as ‘unsafe’ food are in bold text.

### Confidence in being able to avoid foodborne illness

At the same time, many respondents confidently expressed that food could not make them sick – at least if it was the right kind of food (e.g. vegetables) and prepared in the right way (e.g. at home as opposed to in a restaurant, fresh as opposed to leftovers, prepared with lemon and/or vinegar). In some cases, respondents linked the high nutritional content of vegetables to a general healthfulness that encompassed foodborne disease – that is, they knew such foods to be healthy in one way and therefore considered them healthy in all ways. Surprisingly, only about half of Phase 1 respondents reported personal experience with foodborne disease (themselves or family); most of these related to food consumed outside the home (e.g. in a restaurant or at another person’s house). None could recall any food safety-related scares in the community, though they did mention COVID-19 and a bird flu outbreak leading to concerns about certain foods in the market.

Tomato doesn’t make people sick…. The smallest baby and her elder would also eat the raw tomato, and it doesn’t make people sick… [And] I think that kale is very good for health; you won’t get sick from it. I believe that it would protect us from diseases. (Female consumer, 1214)

If kale is cooked well, it is fine… With kale, there is no problem… I have never seen anyone sick [after eating kale]. (Female consumer, 1221)

There is no problem with eating lettuce. Nothing could happen if we could get and eat it. It will protect from disease and doesn’t cause problems. (Male consumer, 1219)

Nothing makes people sick… I have faith that foods do not make people sick. But they might make us sick through our own fault. From lack of hygiene. From deficiencies in preparation and not knowing how to consume… [but] that is from the lack of knowledge, that is not from the food. (Male consumer, 1203)

Similarly, considering food safety as a concern or motivator of food choices, very few consumers brought it up before the topic was raised by the interviewer. However, when asked directly whether food safety was important to them, most replied that it was, citing the potential harm to their family. Some also suggested that having good food quality and/or safety was important due to households’ limited budgets: they did not want or could not afford to have waste or incur expenses for treating illness, as illustrated in the first quote below.

Food safety is something that is crucial to life. If the food is not safe or if we eat a food that is poisoned or a food that does not have safety, we will be attacked by disease. The disease will force us to go to the medical center and that will again be unwanted expense. (Female consumer, 2202)

Well, since we have to eat, we buy from the market. And sometimes I think, I wish, if I ever have enough income I would be happy if I could buy the ones that have been certified with their food safety and that have been packed, because when you go to the market and see on stalks, what they put in there doesn’t look very appealing with its cleanliness…. So it makes me conclude that if I ever can afford it, if I could buy something that has been certified and is safe. (Male consumer, 2237)

Consumers’ generally low level of concern about food safety issues, despite widely recognising their existence, stemmed from their confidence in their ability to choose high-quality foods and to take steps at home to ensure they are safe. The practices consumers reported using to keep foods safe largely aligned to the causes of unsafe food they cited: checking food for signs of damage/insects before buying; washing hands and utensils; washing food; storing food in the refrigerator; cooking food well; eating food promptly after cooking; and disposing of leftovers or thoroughly cooking them before eating. Preparing with lemon and/or vinegar (to ‘kill germs’) was commonly mentioned for lettuce, tomato and other raw vegetables. Within the market, consumers cited looking around to choose ‘the good ones’ and looking for sellers who were in a clean area without dirt, garbage or flies, had arranged their goods attractively and put the vegetables on a clean surface above the ground and covered them. Some also noted looking for a vendor who looked visibly clean, but others claimed this was not important to them. Because food safety issues were seen as largely related to the practices of the food preparer, several respondents also opined that it was dangerous to eat food in restaurants or hotels as opposed to at home, as those places might be less scrupulous in their purchasing and handling choices than the home chef.

### Intermixing quality, safety and freshness

Across all the food safety-related questions, it was clear that respondents did not differentiate between quality more generally and safety, specifically. Replies to questions on safety included comments about quality beyond safety, and comments about quality also were relevant to safety (e.g. being spoiled or damaged by insects). For vegetables specifically, ‘quality’ was closely related to ‘freshness’ (particularly for greens like lettuce) – as one male consumer summarised, ‘If it’s fresh, then that’s good for your health’ (1218). Freshness was indicated by visual appearance (i.e. being crisp-looking and not withered), by visible aspects of handling thought to preserve freshness (e.g. refreshing with water, shading leafy greens) and by the vendors’ assurance that it was newly procured and not left over from a prior day. In general, respondents also tended to equate food safety with other aspects of healthiness – for example, arguing that kale could not cause harm because it was highly nutritious.

Overall, there was not much differentiation among the three focus foods of the study in terms of food safety: consumers saw similar causes and associated illnesses/symptoms among them, with the main distinction being that those eaten raw (lettuce, sometimes tomatoes) were seen as riskier and requiring higher quality standards than those eaten cooked (kale, sometimes tomatoes). For tomatoes, which were eaten both raw and fresh, respondents differentiated between these uses when it came to safety: imperfect tomatoes could cause problems if eaten raw, but not if cooked, so better-quality tomatoes were prioritised for eating raw.

## Discussion

This study has examined the food safety-related perceptions and practices of food consumers in Hawassa, Ethiopia, with a focus on fresh vegetables.

Regarding how consumers conceive of food safety, this study’s results show some contrasts with other work. For example, many other studies have found that consumers in both high- and low/middle-income countries tend to prioritise chemical hazards (e.g. pesticides) over biological hazards such as bacteria^(24–32)^. Here, however, consumers generally worry more about bacterial contamination and issues that could arise through handling, poor cooking or keeping too long as opposed to chemical contamination. This is in line with data from the WHO, which ranks bacteria and viruses (particularly non-typhoidal *Salmonella enterica*) well above chemicals and toxins in terms of driving the burden of foodborne disease, including in Africa^(1)^. In addition, while other studies, including in Ethiopia, have found that consumers tend to identify packaged foods as ‘safer’ than unpackaged ones^(25,31,33)^ and hypothesised this as one pathway through which food safety concerns could have a negative influence on nutrition^(2)^, this was not found here. This is encouraging, as packaged foods tend to be comparatively highly processed and nutrient-poor^(34)^; while experts acknowledge there may be some trade-offs between packaging and safety^(35)^, these can often be mitigated, and packaged foods also pose risks^(36)^. In contrast, the finding that homemade food was perceived as safer than that prepared outside the home aligns with prior research in Asia and Africa^(25,37,38)^, including Ethiopia^(33)^. Whether homemade or non-homemade food is safer is context-specific^(39)^, but as foods consumed outside the home also tend to be higher in calories, salt, sugar and/or fat^(40)^, this finding suggests something of a ‘protective effect’ that food safety worries have on diet quality and nutrition. Food safety being seen as situational is in alignment with some prior findings in LMIC – for example, consumers in urban Ghana were found to prioritise unhygienic settings and visible spoilage (situational aspects) as food safety issues^(30)^ – but not others, for example, a study in Nigeria found that consumers strongly saw certain foods as safer than others, without noting situational differences^(14)^.

One main result is that nearly all respondents had a moderate understanding of at least some of the key aspects of food safety. Food safety was seen as situational, with several main causes of food becoming unsafe: poor handling or storage; food not being properly cooked or eaten raw; spoiled or leftover food; food in restaurants/hotels; and insect damage or infestation. The practices consumers reported using to keep food safe largely aligned with the causes of food safety they cited (e.g. checking food for insects before buying, washing hands and utensils). This understanding generally aligns well with expert food safety guidance^(41)^. Cooking was widely seen to make food safe, with few citing food safety risks that cooking would not eliminate. Moreover, respondents did not clearly differentiate between quality more generally and safety, specifically, and tended to focus on ‘freshness’ as the key marker of quality. Freshness was determined based primarily on visual appearance, using some cues that likely do correlate with safer food (e.g. covering foods) but others (e.g. splashing vegetables with water, well-arranged goods) that may not. This aligns with prior research in Ghana and Nigeria that also found that consumers tend to focus on ‘appearances’ as opposed to actual safety determinants^(42,43)^.

Collectively, these results show a strong foundation of food safety knowledge, aligning with many best practices^(41,44)^ – but also some gaps and misconceptions, such as that cooking and other measures taken at the household level will solve all problems. Knowledge-focused interventions can play a role in correcting these misconceptions and filling these gaps. To maximise impact, these should focus on succinct and memorable messages, such as the WHO’s ‘five keys to safer food’^(41)^, and on specific messages targeted to correct misconceptions, like highlighting that common toxins (e.g. those from Staph bacteria) cannot be killed by cooking.

At the same time, given the high level of existing knowledge, there is clearly a need to move beyond education. This is in contrast to most existing recommendations of prior food safety research in Ethiopia, which focuses primarily on knowledge and training^(8)^. One area on which more focus should be placed is motivating consumers to care about the risks posed by unsafe food. In this study, food safety was not found to be a key driver of market, vendor or food choice for consumers: while they understood the risk of foodborne illness in theory, they generally did not feel personally threatened by it and instead prioritised factors such as price. In general, few consumers reported personally getting sick from food, and most were confident that they could take steps to avoid or mitigate any risk. These results align with prior research showing that food safety is rarely a dominant concern or major driver of food choice among consumers in LMIC^(14,43,45)^. However, they contrast with data showing a high level of foodborne disease risk in Ethiopia and the region^(1,4)^. To close this gap, interventions must focus on increasing the motivation to act – following the ‘wheel’ model of behaviour change^(46)^, this can be done through coercion, incentivization or persuasion. Coercion (e.g. raising the cost of foodborne illness via taxation or fees) is neither feasible nor socially desirable in this case, but incentivization could be used by making consumers more aware of the costs (medical fees, lost wages) they could avoid by prioritising safer food. Education could focus on raising awareness of the food safety risk that exists in Ethiopia, and persuasion could be used to create positive feelings associated with safer food – for example, caring for one’s family or fuelling oneself for work or exercise. Such campaigns could potentially use storytelling (e.g. personal stories of foodborne illness) or gripping visualisations (e.g. indicating the cost of an episode of illness and what else that money could buy) and would likely succeed best through multi-channel distribution (e.g. in market; via radio, posters or billboards). As food safety beliefs are largely shaped by social influences and trust^(37)^, it is essential to ensure that the ‘messenger’ of food safety and nutrition information is a trusted one^(47)^.

Messaging on the topic needs to be developed with care to not frighten consumers counterproductively and dissuade consumption of nutritious foods, which has happened with food safety scares in the past^(48)^. In particular, while vegetables are indeed comparatively highly prone to food safety risks^(2,4)^, with high levels of contamination demonstrated in Ethiopia^(4)^, they are also already under-consumed. Per capita, adult consumption in Ethiopia has been estimated at 48 g/d (compared with the WHO recommended levels of ≥ 240 g/d and the Ethiopia Food-Based Dietary Guidelines recommendation of 100–200 g/d for fruit and vegetables jointly)^(12,49)^. Inadequate consumption of vegetables is a top dietary risk in sub-Saharan Africa^(50)^. It is thus important to mitigate the risk that increased food safety worries lead consumers to avoid vegetables, perhaps substituting with less healthy foods, such as packaged ones^(2,33)^. Such a substitution seems particularly likely given this study’s finding that consumers tend to see nutrition and food safety as interrelated aspects of ‘healthiness’ – and thus might assume a ‘safe’ food is a ‘healthy’ one, even if not nutritious. Mitigating the risk of discouraging vegetable consumption can be done through messaging that integrates nutrition and food safety aspects and is focused on actionable ways to reduce risk. For example, a message that first emphasises the nutritional benefits of consuming vegetables could then include positively framed suggestions of how to select clean vegetables in the market and wash them at home with a focus on ‘making the food even better’, as opposed to presenting vegetables as harmful.

This study has some limitations. First, the sample was small and not statistically representative of the population. This is in line with normal ethnographic research approaches and necessary for the in-depth research approach, which provides a level of detailed insight lacking in most large-sample studies. Data reliability and validity were increased by using a variety of ethnographic techniques and question types, paying careful attention to question ordering and prompting and triangulation. However, the ambiguity of some of the findings may be due to inter-respondent variability, and a large, representative-sample survey could help to contextualise them better. (Indeed, unpublished data collected through a survey in the same market suggested that consumers weighed food safety more highly as a motivator of choice than do these results.) In addition, the consumer sample was drawn from an urban area and is likely more educated and wealthier than a sample from rural Ethiopia would be. Moreover, the study focused on market-based practices, not home food preparation, even though both are key to ensuring food safety. Finally, the focus on raw vegetables omitted both animal-source foods and ready-to-eat foods (known to have considerable food safety challenges). Future research could examine a wider set of foods, focus on home preparation, include rural areas, probe how socio-economic and cultural factors influence food safety perceptions and practices (e.g. through comparative analysis of populations with different socio-economic and/or cultural backgrounds) or consider how broader power structures and forces influence consumers’ choices (and ability to choose) at the local level.

Overall, the results provide a detailed picture of consumer perceptions of food safety in urban Ethiopia, indicating moderate knowledge of the topic but limited worry about it. While encouraging in the sense that they do not indicate food safety functioning as a major barrier to consuming nutritious foods such as vegetables, they contrast with a well-documented risk of foodborne disease in Ethiopia. This underlines the need for innovative interventions that focus not only on educating but on motivating, leveraging not only knowledge but also emotion – and doing so with a focus on nutrition, as well as food safety.
